# Exergy destruction analysis of a power generation system utilizing the cold energy of LNG

**DOI:** 10.1016/j.heliyon.2023.e19393

**Published:** 2023-08-24

**Authors:** Teng Wan, Bin Bai, Weihong Zhou

**Affiliations:** School of Civil Engineering, University of Science and Technology Liaoning, Anshan, 114051, China

**Keywords:** LNG, Exergy analysis, Advanced exergy, Exergy destruction ratio

## Abstract

The purpose of this research is in-depth understanding of the internal causes of exergy destruction in various parts of the system and to identify potential improvements for the components. The focus is on a combined cycle power generation system that utilizes the organic Rankine cycle (ORC) and direct expansion cycle (DEC). To investigate the primary sources of exergy destruction in each component, advanced exergy analysis (AEA) is utilized. The result demonstrates that the net out power of the proposed system can reach 106.64 kW with energy efficiency of 11.22%, and exergy efficiency of 21.40%. The heat exchanger is identified as the primary contributor to exergy destruction, constituting 81.70% of the total ratio. Specifically, the condenser exhibits the highest exergy destruction ratio at 59.82%, indicating a need for prioritized optimization efforts. The findings of AEA reveal that the primary source of component irreversibility stems from the endogenous part. This shows that, while most exergy destruction is unavoidable, there remains room for system improvement. Regarding the turbine, its exergy destruction is primarily attributed to inefficiencies, leading to irreversibility. Nevertheless, there is exergy destruction that may be avoidable and can be reduced by 25.93 kW, which is 2.5 times greater than that of the heat exchanger. This finding underscores the high potential for improvement in ORC and DEC turbines, making them a priority for optimization efforts.

## Highlights

• Comparison between conventional and advanced exergy analysis.
• Each component's exergy destruction is predominantly endogenous.
• 93.89% of the exergy destruction in the condenser is unavoidable.
• The turbine shows significant potential for improvement within the system.

## Nomenclature

*E*x: exergy rate (kW)
*h*: specific enthalpy (kJ/kg)
*m*: mass flow rate (kg/s)
*p*: pressure (MPa)
*Q*: heat (kW)
*s*: specific entropy (kJ/kg·K)
*T*: temperature (°C)
*W*: power (kW)
*y*_D_: exergy destruction ratio
*η*_en_: energy efficiency
*η*_ex_: exergy efficiency
*η*_is_: isentropic efficiency
Δ*T*_min_: minimum temperature difference (°C)

**Greek letters**
*η*: efficiency
Δ: difference
Σ: summation

**Abbreviations**
AEA: advanced exergy analysis
CEA: conventional exergy analysis
COND: condenser
DEC: direct expansion cycle
EVAP: evaporator
HE: heat exchanger
LNG: liquefied natural gas
NG: natural gas
NGH: natural gas heater
ORC: organic Rankine cycle
PU: pump
REH: reheater
TUR: turbine

**Subscripts**
0: ambient condition
cold: cold stream
D: destruction
en: energy
ex: exergy
F: fuel
hot: hot stream
is: isentropic
in: inlet
*k*: the *k*-th component
min: minimum
net: net
out: outlet
P: product
sys: system
S: seawater
tot: total
WF: working fluid

**Superscripts**
AV: avoidable
EN: endogenous
EX: exogenous
UN: unavoidable

## 1 Introduction

It is essential to promote the clean and efficient use of fossil fuels for achieving sustainable development [1,2]. Liquefied natural gas (LNG) as a cryogenic liquid, is produced by deacidifying and dehydrating natural gas (NG) and then compressing and cooling it to −162 °C. However, a significant amount of cold energy is wasted during the LNG gasification process, resulting in low energy utilization [3]. Hence, it is imperative to recuperate the cold energy of LNG prior to its distribution to downstream users.

To improve system performance, current research in this area focuses on improving the cycle structure, optimizing the mixed working medium, selecting different types of heat sources, and utilizing a combined cycle process [[4], [5], [6]]. Wang et al. [7] proposed using LNG cold energy to generate power while also capturing CO_2_. The system's exergy efficiency could reach 56.9%, with the heat exchanger accounting for 94.87% of the system's exergy destruction, according to study. Pan et al. [8] analyzed a cascade utilization system of utilization LNG cold energy. According to their conventional exergy analysis (CEA) results, the heat exchanger and demethanizer accounted for 48.05% and 37.16% of the exergy destruction, respectively. Jeong et al. [9] optimized the mixture medium ratio of ORC using a genetic algorithm, resulting in a 58.4% increase in system power generation.

While many scholars have analyzed cold energy utilization systems, there are still some unresolved issues. CEA can determine the degree and location of exergy destruction, but it is unable to identify the particular sources or improvement potential. On the other hand, AEA is capable of finding the causes of exergy destruction, as well as components with real improvement potential. Morosuk and Tsatsaronis [10,11] first proposed splitting exergy destruction into unavoidable/avoidable and endogenous/exogenous categories and conducted AEA on LNG-based cogeneration systems. Kelly et al. [12] further proposed four methods for calculating endogenous exergy destruction, while Tesch et al. [13] performed CEA and AEA on two air separation system schemes that made use of LNG cold energy. Zhang et al. [14] analyzed a combined system with transcritical CO_2_ energy storage and ORC using CEA and AEA, which provided more reasonable suggestions for optimizing the system. Cai et al. [15] evaluated the efficiency of an oxy-fuel combustion system utilizing LNG cold energy through CEA and AEA and found that improving the flue gas treatment unit in the combustor positively affected system efficiency. Özen et al. [16] applied CEA and AEA to an LNG cold energy power generation system and found that further optimization of the parabolic solar collector and turbine was worthwhile. Additionally, Liu et al. [17] used CEA and AEA to analyze a liquid carbon dioxide energy storage system, emphasizing the importance of turbines in improving system performance.

From the aforementioned literature, it is evident that there are relatively few studies employing AEA in LNG cold energy power generation systems, with a predominant focus on energy and exergy analysis for studies and optimization. Therefore, this study proposes a cold energy power generation system comprising a single-stage ORC and a two-stage DEC. A detailed exergy destruction study is performed on the proposed system based on CEA and AEA in order to identify potential improvements and internal causes of exergy destruction. The study can provide valuable insights for optimizing and designing the power generation process in practical engineering.

## 2 System description

**Fig. 1:**
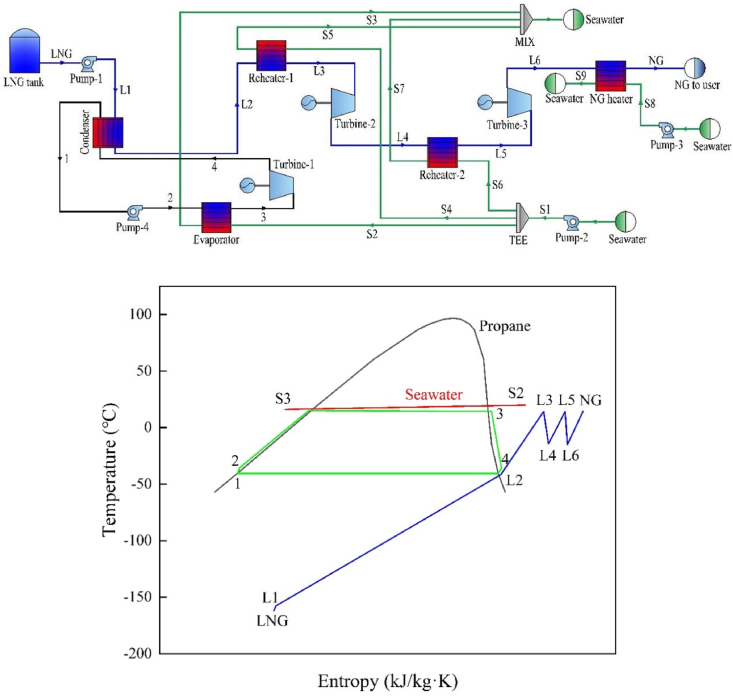
The schematic and T-s diagram of process flow.

The combination of LNG cold energy with ORC has emerged as the predominant method for cold energy generation, offering increased system capacity with the help of increased DEC [18]. In Japan, most LNG cold energy power plants currently utilize ORCs, DECs, and combined cycles to generate power [19]. Fig. 1 illustrates the process of LNG cold energy combined cycle generation, which incorporates an ORC and DEC. The system primarily consists of six components: condenser (COND), evaporator (EVAP), reheater (REH), NG heater (NGH), turbine (TUR), and pump (PU).

In the ORC part of the process, propane undergoes pressurization by PU-4 and is then evaporated into saturated vapor through the evaporator. The saturated vapor is expanded to low pressure in TUR-1, while the resulting exhaust steam from the outlet of TUR-1 returns to the condenser for condensation, thus completing the entire cycle, as depicted in the ORC's T-s diagram. In relation to the DEC part, LNG is subjected to compression at high pressure by PU-1. It is then vaporized in COND, followed by further heat absorption and heating processes in REH-1 and REH-2. Power generation takes place in TUR-2, followed by expansion to the target pressure in TUR-3. Finally, NG is heated to above 0 °C by NGH to be supplied to downstream gas users.

### 2.1 Assumptions and parameter settings

The simulation process is based on the following reasonable assumptions listed below [[20], [21], [22], [23]]：

(1) The system is modeled in steady-state conditions.
(2) The ignoring of pressure drops in heat exchangers and pipelines.
(3) The neglect of heat transfer between components and the surrounding environment.
(4) Kinetic and potential energy effects are disregarded.
(5) Possible chemical reactions within the system are not considered.

To analyze the process, it is necessary to determine the components of LNG, which vary depending on its origin. Table 1 lists the selected components of LNG. Table 2 displays the system's primary parameters. For the goal of simulating the system process, the Aspen HYSYS V11 is used.

**Table 1:** Composition of LNG.

Component	CH_4_	C_2_H_6_	C_3_H_8_	i-C_4_H_10_	n-C_4_H_10_	C_5_H_12_	N_2_
Molar fraction (%)	90.31	5.35	3.21	0.59	0.40	0.03	0.11

**Table 2:** System parameter settings [[24], [25], [26], [27], [28]].

Parameter	Value
*T*_LNG,in_ (°C)	−162.00
*P*_LNG,in_ (MPa)	0.13
*m*_LNG_ (kg/h)	3600
*T*_S,in_ (°C)	20.00
*P*_S,in_ (MPa)	0.10
*T*_0_ (°C)	25
*P*_0_ (MPa)	0.10
Δ*T*_min_ (°C)	5
*η*_is,TUR_ (%)	80
*η*_is,PU_ (%)	80

Moreover, the Peng-Robinson equation of state is chosen as the model for calculating substances' properties [29]. The convergence tolerance for all component models in HYSYS is set to be 1 × 10^−4^.

## 3 Thermodynamic model

### 3.1 Energy analysis

The energy equations for each component are given in equations (1)–(3) based on the first law of thermodynamics [30,31].

(1) $$Q_{\text{HE}}=m{\left(h_{\text{in}}-h_{\text{out}}\right)}_{\text{hot}}=m{\left(h_{\text{out}}-h_{\text{in}}\right)}_{\text{cold}}$$

(2) $$W_{\text{TUR}}=m\left(h_{\text{in}}-h_{\text{out}}\right)$$

(3) $$W_{\text{PU}}=m\left(h_{\text{out}}-h_{\text{in}}\right)$$

Equation (4) can be used to determine the system's net out power (*W*_net_).

(4) $$W_{\text{net}}=\sum W_{\text{TUR}}-\sum W_{\text{PU}}$$

in which, Σ*W*_TUR_ is expressed as the summation of turbine output power; Σ*W*_PU_ is expressed as the summation of pump input power.

Total heat absorption for the system is calculated by equation (5).

(5) $$Q_{\text{tot}}=Q_{\text{EVAP}}+Q_{\text{REH}-1}+Q_{\text{REH}-2}+Q_{\text{NGH}}$$

therefore, the system's energy efficiency is defined by equation (6) [32].

(6) $$\eta_{\text{en},\text{sys}}=\frac{W_{\text{net}}}{Q_{\text{tot}}}\times 100\%$$

### 3.2 Conventional exergy analysis

Using equation (7), we can calculate each state point's exergy rate [33].

(7) $$E_{x}=m\left[\left(h-h_{0}\right)-T_{0}\left(s-s_{0}\right)\right]$$

On the other hand, the exergy destruction of the *k*-th component is determined by equation (8) [34].

(8) $$E_{D,k}=E_{F,k}-E_{P,k}$$

where *E*_F,*k*_ and *E*_P,*k*_ denote the fuel and product exergy, respectively.

Exergy efficiency is commonly employed in exergy analysis to assess the utilization rate of the component. It can be calculated by equation (9) [35].

(9) $$\eta_{\text{ex},k}=\frac{E_{P,k}}{E_{F,k}}\times 100\%$$

By neglecting the fuel exergy of seawater, the system's exergy efficiency is defined according to equation (10) [36].

(10) $$\eta_{\text{ex},\text{sys}}=\frac{W_{\text{net}}}{E_{\text{LNG}}-E_{\text{NG}}}\times 100\%$$

The exergy destruction ratio can indicate the proportion of each link in a system as well as the distribution of it. It is calculated by equation (11) [37].

(11) $$y_{D,k}=\frac{E_{D,k}}{E_{D,\text{tot}}}\times 100\%$$

### 3.3 Advanced exergy analysis

#### 3.3.1 Avoidable and unavoidable exergy destruction

The unavoidable part ($E_{D,k}^{\text{UN}}$) cannot be mitigated due to technical, economic, environmental, and other factors. However, it is feasible to minimize the occurrence of the avoidable part ($E_{D,k}^{\text{AV}}$) by optimizing and improving specific components. The specific formula for this reduction is defined by equation (12) [38].

(12) $$E_{D,k}=E_{D,k}^{\text{AV}}+E_{D,k}^{\text{UN}}$$

The formula for unavoidable exergy destruction is calculated according to equation (13) [39].

(13) $$E_{D,k}^{\text{UN}}=E_{P,k}^{\text{real}}{\left(\frac{E_{D,k}}{E_{P,k}}\right)}^{\text{UN}}$$

in which, $E_{P,k}^{\text{real}}$ is the product exergy in the real condition; ${\left(\frac{E_{D,k}}{E_{P,k}}\right)}^{\text{UN}}$ is under unavoidable condition's exergy destruction to product exergy ratio.

HYSYS simulates the working conditions of components under unavoidable conditions, and the exergy values for each state point are obtained and then substituted into the CEA model. In this manner, avoidable and unavoidable parts can be calculated.

#### 3.3.2 Endogenous and exogenous exergy destruction

AEA can investigate the interaction of various components in more detail. Exergy destruction can be further classified into two types: the endogenous part ($E_{D,k}^{\text{EN}}$), which occurs as a result of the component's own activity, and the exogenous part ($E_{D,k}^{\text{EX}}$), which results from the component's interaction with other components. Hence, exergy destruction can be calculated using equation (14) as shown below [40].

(14) $$E_{D,k}=E_{D,k}^{\text{EN}}+E_{D,k}^{\text{EX}}$$

The system's exergy destruction is calculated from equation (15) [41].

(15) $$E_{D,\text{tot}}=E_{D,k}^{\text{EN}}+E_{D,k}^{\text{EX}}+E_{D,\text{others}}$$

where *E*_D, others_ is the exergy destruction of other components without the *k*-th component.

**Fig. 2:**
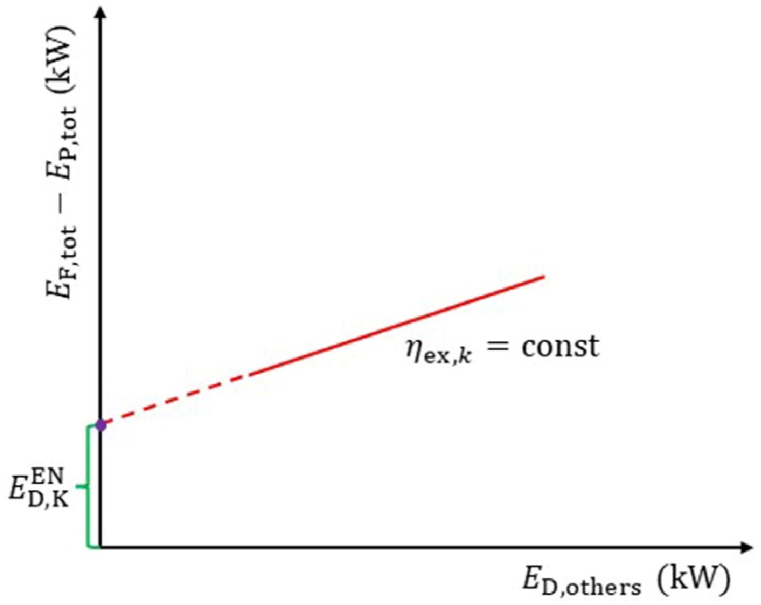
Obtain endogenous exergy destruction by engineering method.

When calculating the endogenous part, linear equations for *E*_F,tot_-*E*_P,tot_ and *E*_D,__others_ need to be plotted as depicted in Fig. 2. By setting *E*_D,__others_s_r_s = 0, the point of intersection between the linear equation and the Y-axis represents the endogenous part. The linear relationship has been validated in the relevant literature. To obtain the linear equations for *E*_F,tot_-*E*_P,tot_ and *E*_D,__others_, at least two sets of data (*E*_F,tot_-*E*_P,tot_, *E*_D__others_) are required. One set can be directly obtained from the results of CEA, while the other set needs to be obtained through the calculation results of using HYSYS for AEA. Furthermore, while calculating a component's endogenous part, the exergy efficiency of the component should correspond to its real operating conditions, and the operating conditions of other components are considered to be ideal.

#### 3.3.3 Exergy destruction that combined four types

The exergy destruction can be further classified into the avoidable endogenous part ($E_{D,k}^{\text{AV},\text{EN}}$), the unavoidable endogenous part ($E_{D,k}^{\text{UN},\text{EN}}$), the avoidable exogenous part ($E_{D,k}^{\text{AV},\text{EX}}$), and the unavoidable exogenous part ($E_{D,k}^{\text{UN},\text{EX}}$) by integrating the four aforementioned methods. For each category can be determined by equations (16)–(21) [42,43].

(16) $$E_{D,k}=E_{D,k}^{\text{AV},\text{EN}}+E_{D,k}^{\text{UN},\text{EN}}+E_{D,k}^{\text{AV},\text{EX}}+E_{D,k}^{\text{UN},\text{EX}}$$

(17) $$E_{P,k}^{\text{EN}}=\frac{E_{D,k}^{\text{EN}}}{1-\eta_{\text{ex},k}}\eta_{\text{ex},k}$$

(18) $$E_{D,k}^{\text{AV},\text{EN}}=E_{D,k}^{\text{EN}}-E_{D,k}^{\text{UN},\text{EN}}$$

(19) $$E_{D,k}^{\text{UN},\text{EN}}=E_{P,k}^{\text{EN}}{\left(\frac{E_{D,k}}{E_{P,k}}\right)}^{\text{UN}}$$

(20) $$E_{D,k}^{\text{AV},\text{EX}}=E_{D,k}^{\text{AV}}-E_{D,k}^{\text{AV},\text{EN}}$$

(21) $$E_{D,k}^{\text{UN},\text{EX}}=E_{D,k}^{\text{UN}}-E_{D,k}^{\text{UN},\text{EN}}$$

where $E_{P,k}^{\text{EN}}$ denotes the endogenous exergy production.

## 4 Model validation

The operating parameters given in Ref. [44] were used to validate the thermodynamic model employed in this study. Table 3 displays a comprehensive comparison of the primary performance parameters between the existing literature and the present research. The table reveals that the performance parameters are generally consistent, with a maximum relative deviation of 1.93%. In most cases, the deviation is less than 2%. It is worth noting that the deviation could be attributed to the utilization of different simulation software. Nonetheless, this supports the possibility of further analysis of the system and validates the reliability of the thermodynamic model used in this research.

**Table 3:** Comparison of ORC results from the reference and the present study.

Parameter	Reference [44]	Present	Deviation (%)
*W*_TUR_ (kW)	114.07	113.3	−0.68
*W*_PU_ (kW)	5.36	5.40	0.75
*Q*_EVAP_ (kW)	1609.40	1630.92	1.34
*Q*_COND_ (kW)	1500.70	1522.99	1.49
*m*_WF_ (kg/s)	4.21	4.21	
*m*_air_ (kg/s)	26.86	26.86	
*W*_net_ (kW)	108.70	107.9	−0.74
*η*_en,sys_ (%)	6.75	6.62	−1.93

## 5 Results and discussion

### 5.1 Design performance

**Fig. 3:**
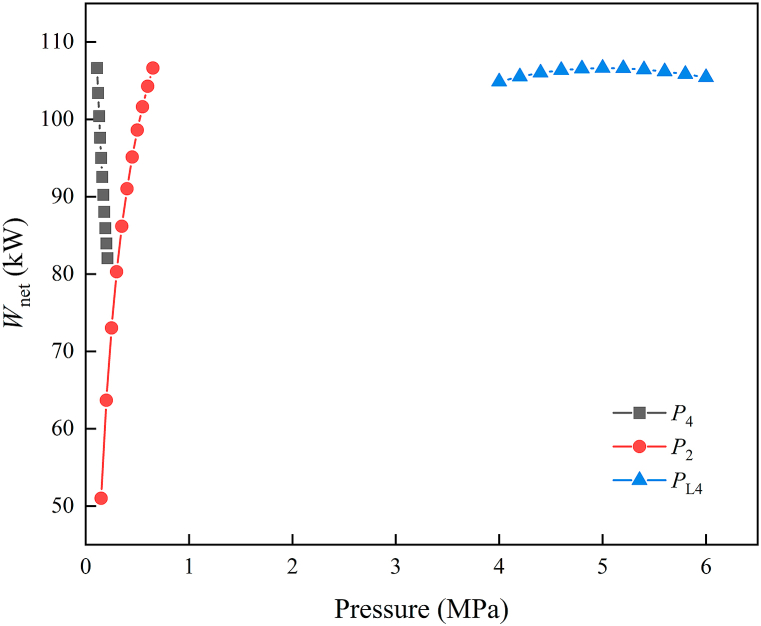
Sensitivity analysis of system's the key parameters.

Condensation pressure (*P*_4_), evaporation pressure (*P*_2_), and first-order expansion pressure (*P*_L4_) were found to significantly affect system performance in a sensitivity analysis of the system's main parameters. As depicted in Fig. 3, increasing *P*_4_ causes a progressive decline in the system's net out power. Similarly, as *P*_2_ grows, the net out power growth trends slow. Furthermore, the system's net out power originally rises and then falls as *P*_L4_ increases, following a parabolic trend. If directly lower LNG pressure to the target pressure by TUR-2, the outlet dryness of TUR-2 is 0.98, and produced liquid drops will cause corrosion to the turbine blades, which is not conducive to components maintenance. Therefore, LNG pressure is lowered to a specific intermediate pressure, and heat exchanges with seawater in REH-2 before entering TUR-3. Finally, expanding to the desired pressure in TUR-3. The parameter of each state point are presented in Table 4. Additionally, Table 5 demonstrates the system's main thermodynamic performance at design conditions.

**Table 4:** The paremeter of each state point.

State point	*T* (°C)	*P* (MPa)	*m* (kg/h)	Vapor fraction
1	−40.30	0.11	4041	0
2	−39.97	0.65	4041	0
3	15.00	0.65	4041	1
4	−36.63	0.11	4041	1
LNG	−162.00	0.13	3600	0
L1	−158.39	8.00	3600	0
L2	−44.38	8.00	3600	1
L3	15.00	8.00	3600	1
L4	−14.39	5.00	3600	1
L5	15.00	5.00	3600	1
L6	−15.00	3.00	3600	1
NG	15.00	3.00	3600	1
S1	20.03	0.50	146020	0
S2	20.03	0.50	91500	0
S3	15.00	0.50	91500	0
S4	20.03	0.50	41500	0
S5	15.00	0.50	41500	0
S6	20.03	0.50	13020	0
S7	15.00	0.50	13020	0
S8	20.03	0.50	11810	0
S9	15.00	0.50	11810	0

**Table 5:** Thermodynamic performance of the system.

Parameter	Value
*W*_TUR-1_ (kW)	71.33
*W*_TUR-2_ (kW)	36.15
*W*_TUR-3_ (kW)	43.29
*W*_PU-1_ (kW)	21.21
*W*_PU-2_ (kW)	19.99
*W*_PU-3_ (kW)	1.62
*W*_PU-4_ (kW)	1.31
*W*_net_ (kW)	106.64
*η*_en, sys_ (%)	11.22
*η*_ex, sys_ (%)	21.40

### 5.2 Simulation results of conventional exergy analysis

**Fig. 4:**
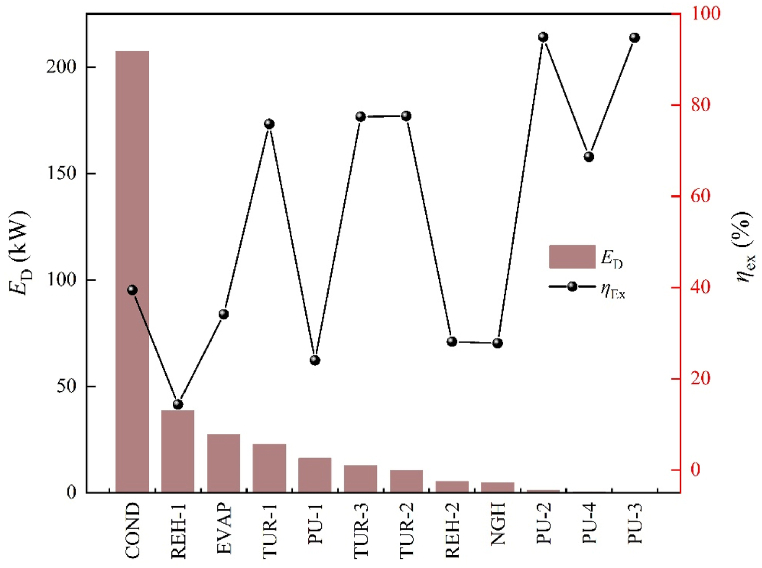
Distribution of exergy destruction and exergy efficiency.

**Fig. 5:**
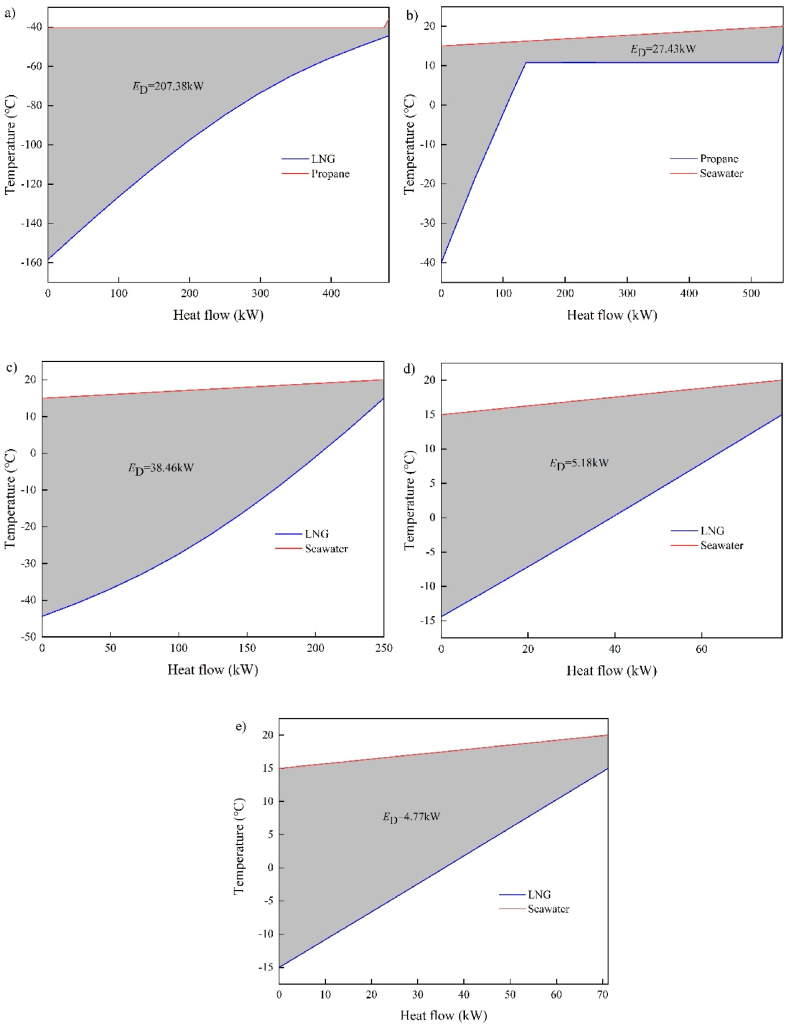
The heat transfer curves in a) COND, b) EVAP, c) REH-1, d) REH-2, e) NGH.

Table 6 displays CEA results. The distribution of exergy destruction and exergy efficiency among the components, as seen in Fig. 4. The heat exchanger exhibits a low exergy efficiency, with the largest exergy destruction (283.21 kW), representing 81.70% of the overall exergy destruction ratio. COND contributes to 59.82% of the exergy destruction, owing to a mismatch between the propane condensation curve and the LNG evaporation curve, as illustrated in Fig. 5(a). The heat transfer process in COND involves a phase change and is hindered by excessive temperature differences, resulting in reduced heat transfer efficiency and significant irreversible destruction. Consequently, COND is the leading cause of exergy destruction. Moreover, as depicted in Fig. 5(b–e), the direct heat transfer with seawater in EVAP, REH-1, REH-2, and NGH results in increased exergy destruction. The turbine and pump exhibit an exergy destruction ratio of 13.21% and 5.08%, respectively. This can be attributed to the inefficient operation of these components, which amplifies exergy destruction. The exergy destruction of PU-2, PU-3, and PU-4, however, is relatively negligible.

**Table 6:** Calculation results of CEA.

Component	*E*_F,*k*_ (kW)	*E*_P,*k*_ (kW)	*E*_D_ (kW)	*η*_ex_ (%)	*y*_D_ (%)
COND	342.20	134.82	207.38	39.40%	59.82%
EVAP	41.64	14.21	27.43	34.13%	7.91%
REH-1	44.90	6.44	38.46	14.35%	11.09%
REH-2	7.20	2.02	5.18	28.11%	1.49%
NGH	6.60	1.83	4.77	27.79%	1.37%
TUR-1	94.08	71.33	22.75	75.82%	6.56%
TUR-2	46.60	36.15	10.45	77.58%	3.01%
TUR-3	55.90	43.29	12.61	77.44%	3.64%
PU-1	21.21	5.10	16.11	24.05%	4.65%
PU-2	19.99	18.97	1.02	94.89%	0.29%
PU-3	1.62	1.53	0.08	94.76%	0.02%
PU-4	1.31	0.90	0.41	68.65%	0.12%

Based on Fig. 4, the priority order for component improvement is as follows: COND > REH-1 > EVAP > TUR-1 > PU-1 > TUR-2 > TUR-3 > REH-2 > NGH > PU-2 > PU-4 > PU-3. This implies that optimizing the COND should be the primary focus for reducing system exergy destruction. It should be noted that this prioritization is a limitation of CEA, which only allows for a quantitative assessment of each component.

### 5.3 Results of advanced exergy analysis

AVA is used to determine the components that require improvement and analyze the interactions between them. Table 7 provides a summary of the thermodynamic data for real, unavoidable, and ideal running conditions.

**Table 7:** Running conditions in real, ideal, and unavoidable [45,46].

Component	Parameter	Real condition	Unavoidable condition	Ideal condition
Condenser	Δ*T*_min_	5 °C	0.5 °C	0 °C
Evaporator	Δ*T*_min_	5 °C	0.5 °C	0 °C
Reheater	Δ*T*_min_	5 °C	0.5 °C	0 °C
NG heater	Δ*T*_min_	5 °C	0.5 °C	0 °C
Turbine	*η*_is_	80%	95%	100%
Pump	*η*_is_	80%	95%	100%

#### 5.3.1 Avoidable and unavoidable exergy destruction

**Fig. 6:**
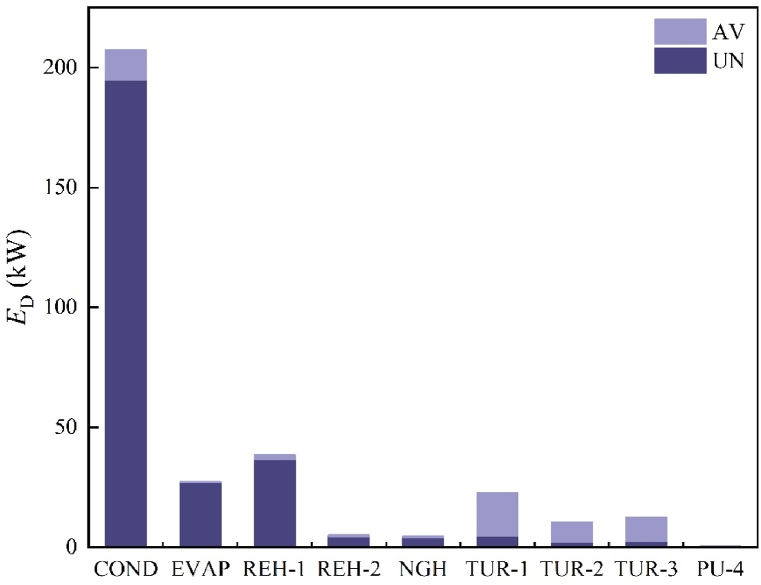
Distribution of avoidable and unavoidable.

The avoidable part can be mitigated by making energy-saving improvements. On the other hand, because of technical or material limits, the unavoidable part cannot be mitigated [47]. Fig. 6 depicts the distribution of avoidable and unavoidable parts among the major components. The avoidable part is the primary determinant of system performance optimization potential. In Fig. 6, the unavoidable part of COND, EVAP, REH-1, REH-2, and NGH is significantly higher than the avoidable part. That is, improving the performance of these components would have little effect on the whole system. However, the avoidable part of TUR-1, TUR-2, and TUR-3's exergy destruction is superior to the unavoidable part, indicating that improving the turbine should be prioritized. This finding aligns with the analysis results reported in other literature [48,49].

#### 5.3.2 Endogenous and exogenous exergy destruction

**Fig. 7:**
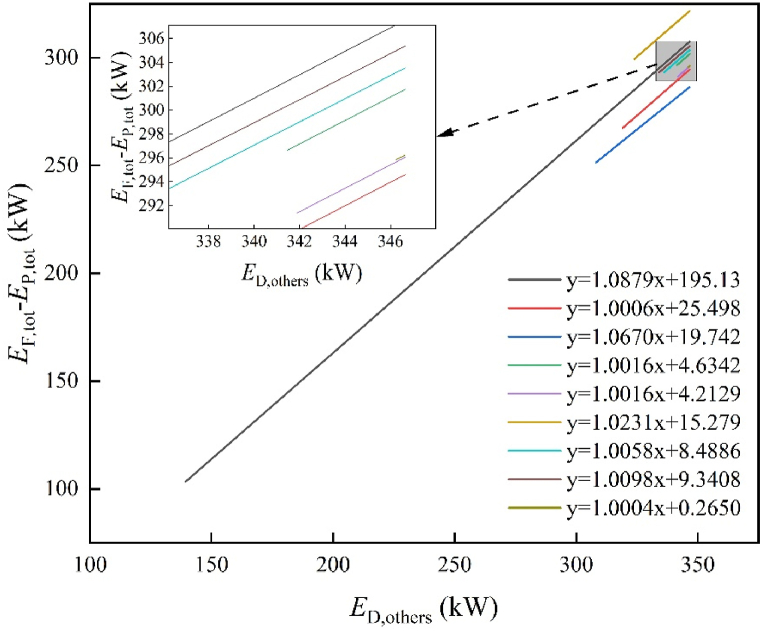
Linear equation diagram.

**Fig. 8:**
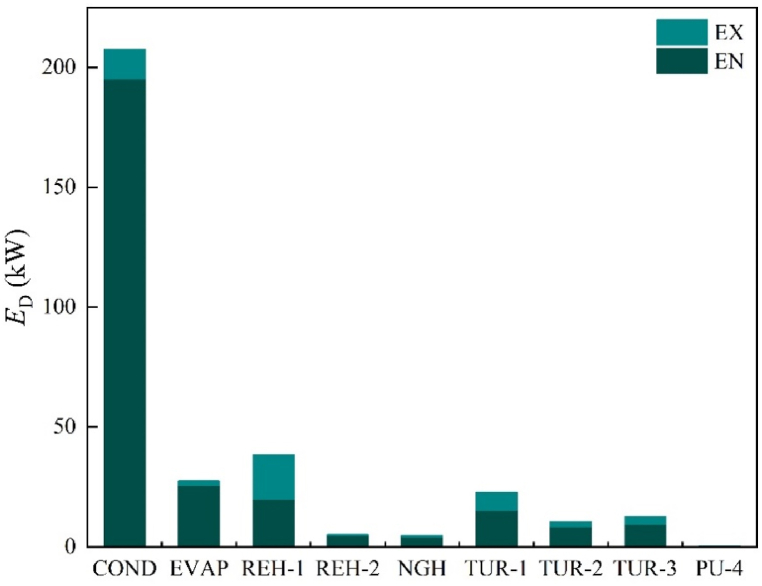
Distribution of endogenous and exogenous.

The sources of exergy destruction and how one component affects the other are revealed by endogenous and exogenous exergy parts [50]. For each component, we calculated the endogenous part using the previously indicated engineering method, and the results are presented in Fig. 7. Next, using equation (9), we computed the exogenous part. Fig. 8 illustrates the endogenous and exogenous parts of the main components. Notably, for each component's exergy destruction, the endogenous part surpasses the exogenous part. This suggests that there is a limited association between the exergy destruction resulting from external factors, such as the system's structure. Additionally, optimizing the design of components and selecting suitable working conditions can further enhance system performance. Consequently, endogenous exergy destruction serves as a crucial parameter in AEA. This enables us to determine the impact of improved components on overall exergy destruction.

#### 5.3.3 Exergy destruction that combined four types

**Fig. 9:**
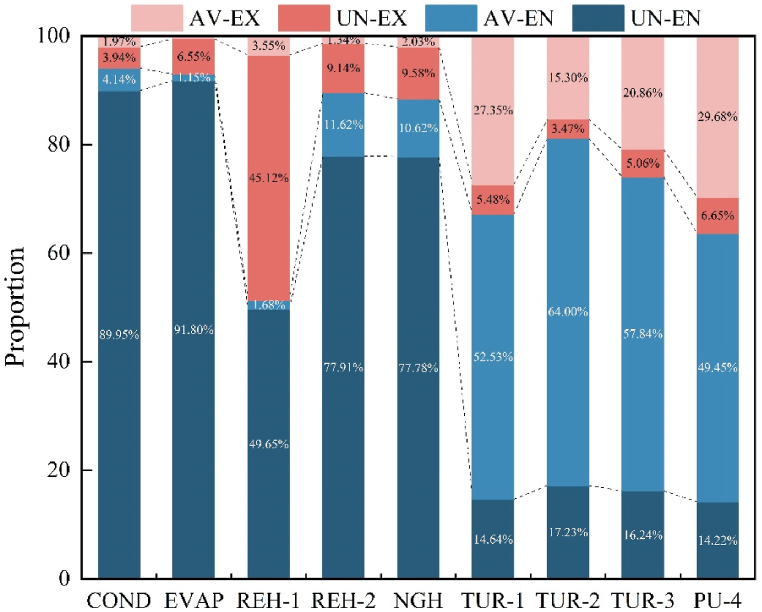
The proportion of four types.

Clarifying the interrelationships between the components necessitates a dependable strategy. Consequently, further research is required on exergy destruction. Table 8 presents the combined exergy destruction for the four types. Simultaneously, the (*E*_D,*k*_/*E*_P,*k*_)^UN^ values obtained during the calculation of the unavoidable part can directly reflect each component's improvement potential. Table 8 displays the (*E*_D,*k*_/*E*_P,*k*_)^UN^ values of the heat exchanger are significantly higher than those of the turbine and pump. Fig. 9 demonstrates the distribution of four distinct categories, which varies from component to component. LNG and propane in COND have a greater temperature difference, resulting in increased exergy destruction. Therefore, COND's unavoidable part accounts for approximately 93.89%. Additionally, although the minimum temperature difference of COND and the influencing factors of the process structure is reduced under unavoidable conditions, the decrease in exergy destruction appears to be very limited, with the avoidable endogenous part accounting for only about 4.14%. This demonstrates the limited optimization potential of COND. Similar situations occur in EVAP, REH-1, REH-2, and NGH, indicating that it may be challenging to reduce the overall system's exergy destruction by improving these components.

**Table 8:** Various types of exergy destruction.

Component	*E*_D_ (kW)								
	${\left(E_{D,k}/E_{P,k}\right)}^{UN}$	$E_{D,k}^{AV}$	$E_{D,k}^{UN}$	$E_{D,k}^{EN}$	$E_{D,k}^{EX}$	$E_{D,k}^{AV,EN}$	$E_{D,k}^{UN,EN}$	$E_{D,k}^{AV,EX}$	$E_{D,k}^{UN,EX}$
COND	1.44	12.67	194.70	195.13	12.25	8.59	186.54	4.08	8.17
EVAP	1.90	0.45	26.98	25.50	1.93	0.32	25.18	0.14	1.79
REH-1	5.66	2.01	36.45	19.74	18.72	0.65	19.10	1.36	17.35
REH-2	2.23	0.67	4.51	4.63	0.54	0.60	4.03	0.07	0.47
NGH	2.27	0.60	4.16	4.21	0.55	0.51	3.71	0.10	0.46
TUR-1	0.07	18.17	4.58	15.28	7.47	11.95	3.33	6.22	1.25
TUR-2	0.06	8.29	2.16	8.49	1.96	6.69	1.80	1.60	0.36
TUR-3	0.06	9.92	2.69	9.34	3.27	7.29	2.05	2.63	0.64
PU-4	0.09	0.33	0.09	0.27	0.15	0.21	0.06	0.12	0.03

In contrast, compared to heat exchangers, the turbine can reduce its own exergy destruction by approximately 78.70%–79.88% or more, with exergy destruction that may be avoidable endogenous part can be reduced by 52.53%–64.00%. This is due to the fact that under unavoidable conditions, the turbine's isentropic efficiency increases from 80% to 95%, causing the enthalpy difference to increase, thereby enhancing power generation and reducing irreversibility. Therefore, by improving the turbine's efficiency to increase system performance, there is a significant improvement in reducing system exergy destruction. Although the isentropic efficiency of PU-4 has also been improved to 95%, it has almost no significant effect on system performance improvement. Furthermore, by adjusting the operational settings of other components and the overall process structure, the turbine's avoidable exogenous part can be diminished. This implies that turbine irreversibility is the principal cause of the system's avoidable exogenous part. Therefore, interactions with other components must be considered in addition to the internal characteristics of the turbine.

According to the results of Table 8, the optimization order is as follows: TUR-1 > COND > TUR-3 > TUR-2 > REH-1 > REH-2 > NGH > EVAP > PU-4. Additionally, the turbine has an avoidable endogenous part of 25.93 kW, which is approximately two and a half times that of the heat exchanger. Therefore, TUR-1 takes priority for optimization, followed by COND, TUR-2, and TUR-3.

#### 5.3.4 Comparison of the results

**Fig. 10:**
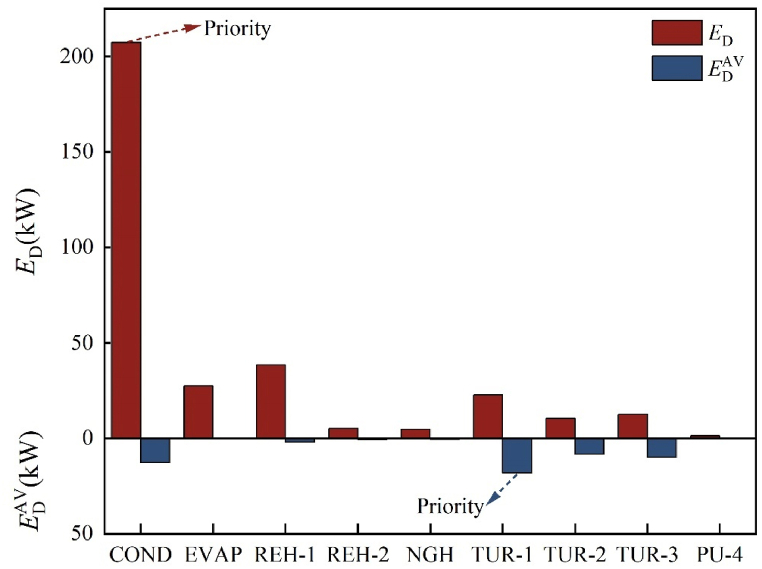
Comparison between CEA and AEA.

The prioritization of the corresponding components serves as a key point of differentiation between the two types of exergy analysis, according to Fig. 10. Since COND exhibits the highest exergy destruction (207.38 kW), CEA recommends optimizing this component first. However, AEA reveals a significant proportion of exergy destruction is unavoidable, emphasizing the need to prioritize TUR-1, which has the greatest amount of avoidable exergy destruction (18.17 kW). Therefore, when conducting a thermodynamic analysis of the system, it is essential not only to consider CEA methods but also to incorporate AEA methods to acquire an in-depth understanding of the optimization potential of each component and the internal factors contributing to exergy destruction.

## 6 Conclusions

The system's exergy destruction was analyzed using CEA and AEA, which helped identify the optimization direction for components, revealing their improvement potential and playing a critical part in system optimization. The following significant findings were obtained.

1. At base-case conditions, the net power out of the proposed system is 106.64 kW, and the energy efficiency and exergy efficiency can reach 11.22% and 21.40%, respectively.
2. Based on CEA, heat exchangers contributed 81.70% to the total exergy destruction ratio, while turbines and pumps contributed 13.21% and 5.08%. This indicates that heat exchangers contribute the most to exergy destruction, with the condenser having the highest exergy destruction ratio of 59.82%. Therefore, enhancing the condenser's performance should be prioritized.
3. Exergy destruction of heat exchangers was further analyzed using AEA and found to be mainly unavoidable, with improvements to heat exchangers not yielding appreciable gains in system performance. In addition, exergy destruction in components results primarily from their own irreversibility.
4. The potential for improvement in turbines presents an opportunity to enhance system performance. By reducing the avoidable endogenous part, the turbines can contribute to a decrease of 25.93 kW, which is two and a half times greater than that achievable through improving heat exchangers. Therefore, optimizing the turbines in ORC and DEC during system design and optimization is crucial.

In conclusion, AEA results provide valuable supplementary information to CEA findings. It should be stressed, however, that the current study relies on steady-state simulation, which cannot accurately determine component performance under real-time operating conditions, considering factors such as gas transmission volume at LNG terminal and system component maintenance. Additionally, the study only examined the system's exergy-based optimization analysis. Therefore, future research should consider studying the feasibility of the system using dynamic simulation of HYSYS, while incorporating economic and environmental analyses.

## Author contribution statement

Teng Wan: Conceived and designed the experiments; Performed the experiments; Analyzed and interpreted the data; Wrote the paper.

Bin Bai: Performed the experiments; Analyzed and interpreted the data; Wrote the paper.

Weihong Zhou: Analyzed and interpreted the data; Contributed reagents, materials, analysis tools or data.

## Data availability statement

Data will be made available on request.

## Declaration of competing interest

The authors declare that they have no known competing financial interests or personal relationships that could have appeared to influence the work reported in this paper.

## References

[1] Gómez M.R., Gómez J.R., López-González L.M., López-Ochoa L.M. (2016). Thermodynamic analysis of a novel power plant with LNG (liquefied natural gas) cold exergy exploitation and CO_2_ capture. Energy.

[2] Balli O., Ozbek E., Ekici S., Midilli A., Karakoc T.H. (2021). Thermodynamic comparison of TF33 turbofan engine fueled by hydrogen in benchmark with kerosene. Fuel.

[3] Yao S., Yang Y., Zhang Z., Wei Y., Sun J. (2022). Design and optimization of LNG-powered ship cold energy and waste heat integrated utilization system based on novel intermediate fluid vaporizer. Case Stud. Therm. Eng..

[4] Bao J., Lin Y., Zhang R., Zhang X., Zhang N., He G. (2018). Performance enhancement of two-stage condensation combined cycle for LNG cold energy recovery using zeotropic mixtures. Energy.

[5] Xu J., Lin W. (2017). A CO_2_ cryogenic capture system for flue gas of an lng-fired power plant. Int. J. Hydrogen Energy.

[6] Sun Z., Zhao Q., Wu Z., Lin K. (2021). Thermodynamic comparison of modified Rankine cycle configurations for LNG cold energy recovery under different working conditions. Energy Convers. Manag..

[7] Wang X., Zhao L., Zhang L., Zhang M., Dong H. (2019). A novel combined system for LNG cold energy utilization to capture carbon dioxide in the flue gas from the magnesite processing industry. Energy.

[8] Pan J., Li M., Li R., Tang L., Bai J. (2022). Design and analysis of LNG cold energy cascade utilization system integrating light hydrocarbon separation, organic Rankine cycle and direct cooling. Appl. Therm. Eng..

[9] Jeong M., Cho E.B., Byun H.S., Kang C.H. (2021). Maximization of the power production in LNG cold energy recovery plant via genetic algorithm. Kor. J. Chem. Eng..

[10] Morosuk T., Tsatsaronis G. (2008). A new approach to the exergy analysis of absorption refrigeration machines. Energy.

[11] Morosuk T., Tsatsaronis G. (2011). Comparative evaluation of LNG – based cogeneration systems using advanced exergetic analysis. Energy.

[12] Kelly S., Tsatsaronis G., Morosuk T. (2009). Advanced exergetic analysis: approaches for splitting the exergy destruction into endogenous and exogenous parts. Energy.

[13] Tesch S., Morosuk T., Tsatsaronis G. (2016). Advanced exergy analysis applied to the process of regasification of LNG (liquefied natural gas) integrated into an air separation process. Energy.

[14] Zhang Y., Liang T., Yang C., Zhang X., Yang K. (2020). Advanced exergy analysis of an integrated energy storage system based on transcritical CO_2_ energy storage and Organic Rankine Cycle. Energy Convers. Manag..

[15] Cai L., Fu Y., Cheng Z., Xiang Y., Guan Y. (2022). Advanced exergy and exergoeconomic analyses to evaluate the economy of LNG oxy-fuel combined cycle power plant. J. Environ. Chem. Eng..

[16] Özen D.N., Koçak B. (2022). Advanced exergy and exergo-economic analyses of a novel combined power system using the cold energy of liquefied natural gas. Energy.

[17] Liu Z., Liu Z., Yang X., Zhai H., Yang X. (2020). Advanced exergy and exergoeconomic analysis of a novel liquid carbon dioxide energy storage system. Energy Convers. Manag..

[18] Franco A., Casarosa C. (2015). Thermodynamic analysis of direct expansion configurations for electricity production by LNG cold energy recovery. Appl. Therm. Eng..

[19] Gómez M.R., Garcia R.F., Gómez J.R., Carril J.C. (2014). Review of thermal cycles exploiting the exergy of liquefied natural gas in the regasification process. Renew. Sust. Energ. Rev..

[20] Wang J., Wang J., Dai Y., Zhao P. (2014). Thermodynamic analysis and optimization of a transcritical CO_2_ geothermal power generation system based on the cold energy utilization of LNG. Appl. Therm. Eng..

[21] Atienza-Márquez A., Bruno J.C., Coronas A. (2021). Regasification of liquefied natural gas in satellite terminals: techno-economic potential of cold recovery for boosting the efficiency of refrigerated facilities. Energy Convers. Manag..

[22] Saleh B. (2018). Energy and exergy analysis of an integrated organic Rankine cycle-vapor compression refrigeration system. Appl. Therm. Eng..

[23] Chen K., Yu H., Fan G., Zhang Y., Dai Y. (2022). Multi-objective optimization of a novel combined parallel power generation system using CO_2_ and N_2_ for cascade recovery of LNG cryogenic energy. Energy Convers. Manag..

[24] Bao J., Yuan T., Zhang L., Zhang N., He G. (2019). Comparative study of liquefied natural gas (LNG) cold energy power generation systems in series and parallel. Energy Convers. Manag..

[25] Qi M., Park J., Kim J., Lee I., Moon I. (2020). Advanced integration of LNG regasification power plant with liquid air energy storage: enhancements in flexibility, safety, and power generation. Appl. Energy.

[26] Ma G., Lu H., Cui G., Huang K. (2018). Multi-stage Rankine cycle (MSRC) model for LNG cold-energy power generation system. Energy.

[27] Ayou D.S., Eveloy V. (2020). Energy, exergy and exergoeconomic analysis of an ultra low-grade heat-driven ammonia-water combined absorption power-cooling cycle for district space cooling, sub-zero refrigeration, power and LNG regasification. Energy Convers. Manag..

[28] Küçük E.Ö., Kılıç M. (2023). Exergoeconomic analysis and multi-objective optimization of ORC configurations via Taguchi-Grey Relational Methods. Heliyon.

[29] Alabdulkarem A., Mortazavi A., Hwang Y., Radermacher R., Rogers P. (2011). Optimization of propane pre-cooled mixed refrigerant LNG plant. Appl. Therm. Eng..

[30] Han F., Wang Z., Ji Y., Li W., Sunden B. (2019). Energy analysis and multi-objective optimization of waste heat and cold energy recovery process in LNG-fueled vessels based on a triple organic Rankine cycle. Energy Convers. Manag..

[31] Ma J., Song X., Zhang B., Mao N., He T. (2022). Optimal design of dual-stage combined cycles to recover LNG cold energy and low-temperature waste thermal energy for sustainable power generation. Energy Convers. Manag..

[32] Domingues A., Matos H.A., Pereira P.M. (2022). Novel integrated system of LNG regasification/electricity generation based on a cascaded two-stage Rankine cycle, with ternary mixtures as working fluids and seawater as hot utility. Energy.

[33] Balli O., Ekici S., Karakoc H.T. (2021). Achieving a more efficient and greener combined heat and power system driven by a micro gas turbine engine: issues, opportunities, and benefits in the presence of thermodynamic perspective. Int. J. Energy Res..

[34] Güleryüz E.H., Özen D.N. (2022). Advanced exergy and exergo-economic analyses of an advanced adiabatic compressed air energy storage system. J. Energy Storage.

[35] Yang D., Jie Z., Zhang Q., Li Y., Xie J. (2022). Evaluation of the ejector two-stage compression refrigeration cycle with work performance from energy, conventional exergy and advanced exergy perspectives. Energy Rep..

[36] He T., Ma H., Ma J., Mao N., Liu Z. (2021). Effects of cooling and heating sources properties and working fluid selection on cryogenic organic Rankine cycle for LNG cold energy utilization. Energy Convers. Manag..

[37] Tian Z., Qi Z., Gan W., Tian M., Gao W. (2022). A novel negative carbon-emission, cooling, and power generation system based on combined LNG regasification and waste heat recovery: energy, exergy, economic, environmental (4E) evaluations. Energy.

[38] Hamedi M., Omidkhah M., Sadrameli S.M., Manesh M.H.K. (2022). Exploring the improvement potentials in an existing industrial olefin plant through advanced exergy-based analyses. J. Clean. Prod..

[39] Muhammad H.A., Lee B., Cho J., Rehman Z., Choi B., Cho J., Baik Y.J. (2021). Application of advanced exergy analysis for optimizing the design of carbon dioxide pressurization system. Energy.

[40] Zhong X., Chen T., Sun X., Song J., Zeng J. (2022). Conventional and advanced exergy analysis of a novel wind-to-heat system. Energy.

[41] Ansarinasab H., Mehrpooya M., Mohammadi A. (2017). Advanced exergy and exergoeconomic analyses of a hydrogen liquefaction plant equipped with mixed refrigerant system. J. Clean. Prod..

[42] Li L., Liu Z., Deng C., Ren J., Ji F., Sun Y., Yang S. (2021). Conventional and advanced exergy analyses of a vehicular proton exchange membrane fuel cell power system. Energy.

[43] Zhang S., Jing J., Jiang H., Qin M., Chen D., Chen C. (2020). Advanced exergy analyses of modified ethane recovery processes with different refrigeration cycles. J. Clean. Prod..

[44] Xia W., Huo Y., Song Y., Han J., Dai Y. (2019). Off-design analysis of a CO_2_ Rankine cycle for the recovery of LNG cold energy with ambient air as heat source. Energy Convers. Manag..

[45] Bai T., Yu J., Yan G. (2016). Advanced exergy analyses of an ejector expansion transcritical CO_2_ refrigeration system. Energy Convers. Manag..

[46] Mohammadi Z., Fallah M. (2023). Comparative investigation of six layouts of S-CO_2_ cycles based on advanced exergy analysis. Energy Convers. Manag..

[47] Hu X., Liu Y., Dong S., Li G., Sun Z., Liu Y. (2022). Comparison study of conventional and advanced exergy analysis on cascade high temperature heat pump system based on experiment. Case Stud. Therm. Eng..

[48] Nami H., Nemati A., Fard F.J. (2017). Conventional and advanced exergy analyses of a geothermal driven dual fluid organic Rankine cycle (ORC). Appl. Therm. Eng..

[49] Tian Z., Chen X., Zhang Y., Gao W., Chen W., Peng H. (2023). Energy, conventional exergy and advanced exergy analysis of cryogenic recuperative organic rankine cycle. Energy.

[50] Caliskan H., Açıkkalp E., Takleh H.R., Zare V. (2023). Advanced, extended and combined extended-advanced exergy analyses of a novel geothermal powered combined cooling, heating and power (CCHP) system. Renew. Energy.

